# Post-COVID Surge in Pediatric Emergency Department Accesses for Psychiatric Conditions: A Retrospective Analysis of Anxiety, Self-Injury Behaviors, and Psychomotor Agitation

**DOI:** 10.3390/jcm14144814

**Published:** 2025-07-08

**Authors:** Tommaso Bellini, Silvia Merlo, Andrea Lacovara, Sara Uccella, Martino Diana, Martina Turone, Carolina Viglietti, Barbara Tubino, Lino Nobili, Pasquale Striano, Emanuela Piccotti, Andrea Moscatelli, Laura Siri

**Affiliations:** 1 Pediatric Emergency Room and Emergency Medicine Unit, IRCCS Istituto Giannina Gaslini, Via G. Gaslini, 5, 16147 Genoa, Italy; barbaratubino@gaslini.org (B.T.); emanuelapiccotti@gaslini.org (E.P.); 2 Department of Neuroscience, Rehabilitation, Ophthalmology, Genetics, Maternal and Child Health (DINOGMI), University of Genoa, 16146 Genoa, Italy; 4137618@studenti.unige.it (S.M.); 5727889@studenti.unige.it (A.L.); sara.uccella@unige.it (S.U.); 4042985@studenti.unige.it (M.D.); 3220801@studenti.unige.it (M.T.); 4201418@studenti.unige.it (C.V.); linonobili@gaslini.org (L.N.); pasqualestriano@gaslini.org (P.S.); 3 Child Neuropsychiatry Unit, IRCCS Istituto Giannina Gaslini, 16147 Genoa, Italy; laurasiri@gaslini.org; 4 Pediatric Neurology and Neuromuscular Diseases Unit, IRCCS Istituto Giannina Gaslini, 16147 Genoa, Italy; 5 Pediatric and Neonatal Intensive Care Unit, IRCCS Istituto Giannina Gaslini, 16147 Genoa, Italy; andreamoscatelli@gaslini.org

**Keywords:** anxiety disorders, COVID-19 pandemic, emergency department, mental health crisis, psychiatric emergencies, psychomotor agitation, self-harm, self-injury behavior

## Abstract

**Background:** The COVID-19 pandemic has had a profound impact on pediatric mental health, contributing to a global surge in psychiatric emergencies among children and adolescents. This study aimed to evaluate trends in pediatric emergency department (PED) visits for three key psychiatric conditions—anxiety disorders (ADs), self-injury behaviors (SIBs), and psychomotor agitation (PMA)—before and after the onset of the COVID-19 pandemic. **Methods**: We conducted a retrospective observational study at a tertiary pediatric hospital in Italy, analyzing all psychiatric presentations to the PED from 1 January 2018 to 31 December 2024. The data were divided into pre-COVID and post-COVID periods and included patient demographics, recurrence of visits, clinical features, hospital admissions, and pharmacological management. Diagnoses were confirmed by chart review. **Results**: Of 233,867 total PED visits, 1082 were due to primary psychiatric concerns. A marked increase in visits was observed postCOVID: SIB incidence rose from 3.6 to 15.1 per 10,000 visits (*p* < 0.0001), PMA from 9.4 to 17.8 (*p* < 0.0001), and AD from 17.7 to 21.6 (*p* = 0.018). SIB cases showed increased recurrence (from 3.4% to 27.4%, *p* = 0.004) and greater pharmacological intervention, whereas PMA was associated with a rise in heteroaggression (from 14.3% to 39.8%, *p* < 0.0001). Pharmacological treatment remained largely consistent, with benzodiazepines and neuroleptics most frequently used. The emerging use of intranasal ketamine was noted in select cases. **Conclusions**: This study highlights the increasing burden of pediatric psychiatric emergencies in the wake of the COVID-19 pandemic. The findings underscore the urgent need to implement standardized emergency care protocols, strengthen outpatient mental health services, and develop pediatric-specific pharmacological guidelines to improve outcomes in this vulnerable population.

## 1. Introduction

The COVID-19 pandemic has profoundly impacted global mental health, with the pediatric population facing unique and under-recognized challenges [1,2,3,4,5,6]. Measures undertaken to minimize contagion, such as school closures, social distancing, and lockdowns, have been implemented worldwide, leading children to experience unprecedented disruptions to their routines and social development [7]. Children and adolescents face prolonged isolation, familial stress, increased exposure to domestic conflict, and the loss of protective environments, such as school or sports activities, all contributing to heightened psychological distress [1,7]. All of these factors may have contributed to the mental health crisis [2,7,8,9]. Multiple international reports have described a significant increase in pediatric emergency department (PED) accesses for psychiatric reasons among children and adolescents during and after the pandemic, which now constitute one of the fastest-growing categories of PED utilization [7,9,10,11]. Anxiety disorders (ADs), self-injury behaviors (SIBs), including self-harm, suicidal ideation, and suicide attempts, and psychomotor agitation (PMA) have emerged as three prominent presentations during the post-pandemic period, reflecting an increase in mental health burden and the complex interplay of psychosocial stressors exacerbated by the pandemic [1,2,3,4,6,7,8,10,11,12].

Up to 20% of children worldwide are estimated to experience a mental health disorder annually, the prevalence of which has been steadily rising over the past decade [6,7,9]. The reduction in outpatient psychiatric resources, compounded by school closures and social isolation during lockdown periods, has redirected many pediatric psychiatric emergencies to PEDs, which has become a critical entry point for pediatric psychiatric care [2,6,7,8,9].

This trend has been corroborated by national data demonstrating a constant annual rise in pediatric psychiatric ED visits over the past decade, with sharp increases noted during and after the COVID-19 lockdowns [2,11,12].

Moreover, there is a worrying increase in the rate of PED revisits among discharged psychiatric patients due to limited access to follow-up care and persistent social stressors [6,7,10,11,13]. These effects are particularly visible in pediatric emergency settings, where clinicians are increasingly faced with managing complex psychiatric cases without adequate psychiatric resources [1,2,9,11,14].

Compounding this crisis is the limited availability of inpatient psychiatric beds and child/adolescent psychiatrists [2]. These conditions not only pose diagnostic and therapeutic challenges, but also have operational implications for PEDs, contributing to longer lengths of stay, higher use of pharmacological management, and more frequent inpatient psychiatric admissions [8,9,12,13,15]. This phenomenon is known as “psychiatric boarding,” in which children remain in the PED for extended periods awaiting placement [8,11,12]. Studies also show that up to half of pediatric psychiatric patients receive their first mental health evaluation in the emergency department, highlighting the crucial role of PED as a point of entry into the mental health care continuum [6,10,12].

This study aimed to retrospectively analyze PED visits for these three psychiatric conditions (AD, SIB, and PMA) over a five-year period spanning the COVID-19 pandemic. By evaluating trends, clinical features, and management strategies, including pharmacological interventions, we aim to contribute data to inform emergency protocols and improve pediatric mental health care delivery.

## 2. Materials and Methods

This retrospective observational color study was conducted at the PED of Istituto di Ricerca e Cura a Carattere Scientifico (IRCCS) Giannina Gaslini, a tertiary-level pediatric hospital located in Genoa, Italy. The study period spannedfrom 1 January 2018 to 31 December 2024, encompassing both the pre-pandemic and post-pandemic periods. As it was the day the lockdown ended in Italy, 4 May 2020 was chosen as the day of division between the two group,. The PED handles approximately 38,000 accesses annually and maintains a dedicated child psychiatry consultation team for patients aged <15 years, on behalf of a regional sanction. The hospital follows standardized triage and diagnostic protocols for mental health evaluations. Visits were categorized into two periods: preCOVID (1 January 2018 to 4 May 2020) and postCOVID (5 May 2020 to 31 December 2024). Psychiatric-related visits were identified through diagnostic coding and clinical documentation.

We focused on these three core psychiatric presentations in the PED: AD, including generalized anxiety, panic attacks, and acute emotional distress; SIB, encompassing non-suicidal self-injury including cutting, suicide attempts, and related behaviors such as mood swings; and PMA, characterized by severe restlessness, impulsivity, aggression, and physical agitation often requiring sedation.

The inclusion criteria were patients aged <15 years, presentation to the PED with a primary psychiatric concern, and completion of a formal psychiatric consultation. The specific diagnostic categories included AD, SIB, and PMA. Exclusion criteria were incomplete electronic medical records and concurrent life-threatening medical conditions.

Data were extracted from the hospital electronic medical records. Manual chart reviews were performed to validate the psychiatric diagnoses, medications administered, and clinical outcomes. Demographic variables, including age and gender, were extracted from electronic medical records. Clinical outcomes, including hospital admission, use of pharmacological treatment, and presence of co-occurring associated symptoms (heteroaggression, mood instability, or suicide attempts), were also collected when available in the psychiatric assessment. The incidence rates were calculated per 10,000 PED visits. Recurrent PED use was defined as ≥2 visits for psychiatric reasons during the study period and extracted from longitudinal chart linkage. Pharmacological agents were classified by pharmacological class (benzodiazepines, neuroleptics, alpha-2 agonists, and dissociatives). Clinical indications, dosing strategies, routes of administration, and adverse events were recorded when available.

### 2.1. Statistical Analysis

Descriptive statistics were used to summarize the patient demographics and clinical characteristics. Continuous variables are presented as medians with interquartile ranges (IQR) and categorical variables as frequencies and percentages. Chi-square or Fisher’s exact tests were used for categorical comparisons, and Mann–Whitney U tests were used for non-parametric continuous data. Statistical significance was defined as *p* < 0.05. All analyses were performed using SPSS v26 (IBM Corp., New York, NY, USA).

### 2.2. Ethical Considerations

The study was conducted in accordance with the Declaration of Helsinki and approved by the Internal Review Board (n.561 06 July 2021).

## 3. Results

**Figure 1:**
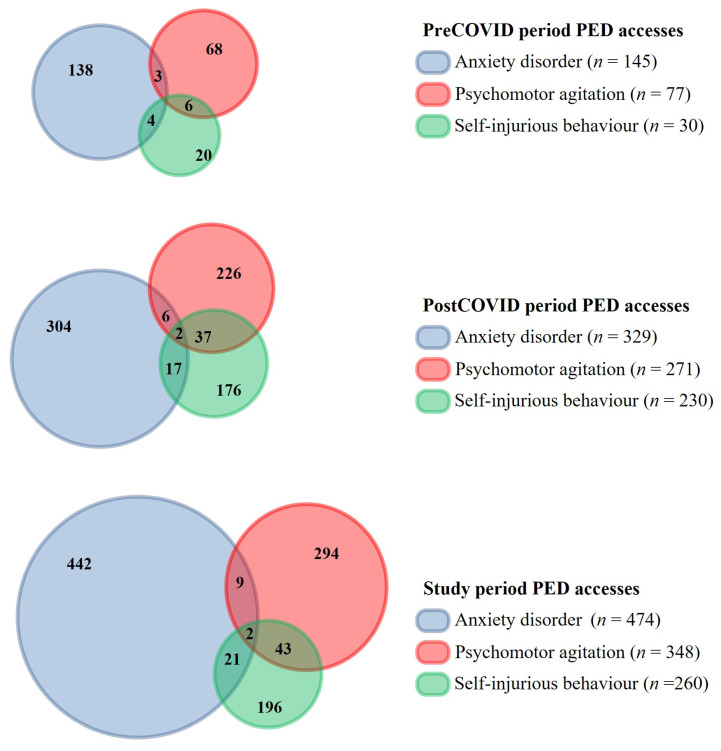
Venn diagrams illustrating the overlap of pediatric emergency department (PED) visits for anxiety disorders (ADs), psychomotor agitation (PMA), and self-harming behavior (SHs) across the three time periods. Each circle represents the total number of visits for a specific mental health condition: blue for anxiety disorders, red for psychomotor agitation, and green for self-harming behavior. The top diagram shows the distribution during the pre-COVID period, with limited overlap among the three conditions. The middle diagram displays data from the post-COVID period, highlighting a marked increase in both the number and overlap of cases. The bottom diagram summarizes the entire study period, showing substantial comorbidities among the three conditions.

A total of 233,867 PED accesses were reviewed, and all pediatric patients aged 0–15 years who presented to the PED with primary psychiatric complaints were included: 81,560 in the pre-COVD period and 152,307 in the post-COVID period. A total of 1082 accesses were identified out of a total of 923 patients. Figure 1 shows a representation of the symptoms and their intersection in both periods. A significant proportion of accesses (n = 75.7%) were due to symptoms that spanned at least two diagnostic categories. For greater clarity, the results were divided into subsections for each type of main psychiatric symptom.

### 3.1. Anxiety Disorders

**Figure 2:**
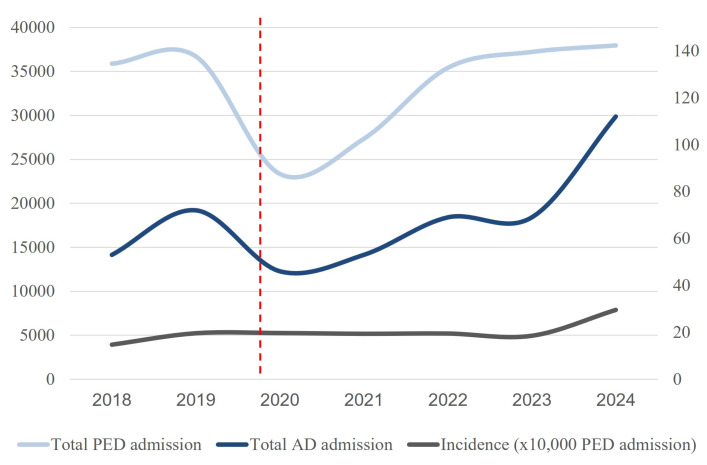
Trends in pediatric emergency department (PED) admissions for anxiety disorders (ADs), total PED visits, and incidence rates over time. The light blue line represents the total number of PED admissions between 2018 and 2024. The dark blue line indicates the total number of visits for anxiety disorders. The gray line shows the incidence of ADs, expressed as the number of cases per 10,000 PED admissions. The vertical red dashed line marks the beginning of the COVID-19 pandemic (2020), after which a notable increase in AD-related admissions and incidences was observed.

A total of 474 accesses for AD were counted, with 145 occurring preCOVID and 329 postCOVID. The incidence increased significantly from 17.7 to 21.6 per 10,000 visits (*p* = 0.018). The median age remained stable (12.5 years), with no significant gender differences across the periods. The hospital admission rates (14.5% vs. 16.1%) and pharmacological interventions (14.5% vs. 14.3%) showed no significant changes. The recurrence of PED visits decreased slightly postCOVID (11.8% vs. 9.8%; *p* = 0.51). Table 1 reports the main results, and Figure 2 shows the trend of PED access and its incidence during the years of the study period.

### 3.2. Self-Injury Behaviors

**Figure 3:**
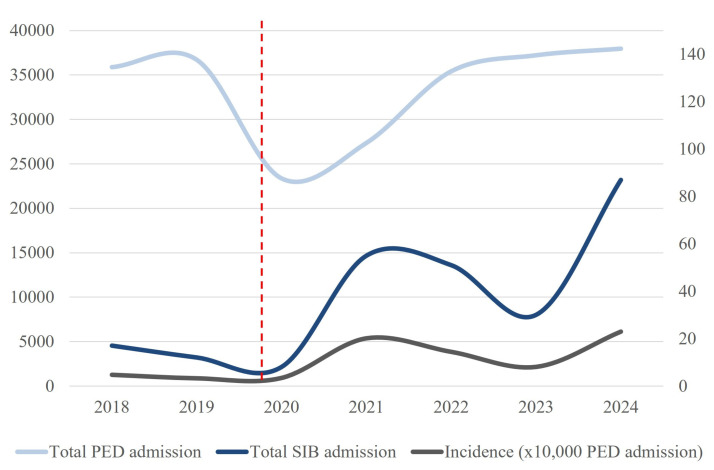
Trends in pediatric emergency department (PED) admissions for self-injurious behavior (SIB), total PED visits, and incidence rates over time. The light blue line represents the total number of PED admissions from 2018 to 2024. The dark blue line indicates the total number of visits related to self-injurious behavior (SIB). The gray line denotes the incidence of SIB, expressed as the number of cases per 10,000 PED admissions. The vertical red dashed line marks the onset of the COVID-19 pandemic (2020), highlighting a significant increase in SIB-related admissions and incidence rates in subsequent years despite the overall reduction in total PED visits.

In total, 260 accesses for SIB were counted. There was a sharp increase in SIB cases from 30 to 230 post-COVID. The incidence significantly surged from 3.6 to 15.1 per 10,000 visits (*p* < 0.0001). Median age increasedslightly (13.2 to 13.6 years; *p* = 0.01), while the proportion of male patients dropped significantly (23.3% to 10.8%; *p* = 0.05). Hospital admission remained consistently high during both periods. Pharmacological treatment increased (*p* = 0.03). Recurrence increased markedly (3.4–27.4%; *p* = 0.004). Concomitant cutting and suicide attempts were present in 33.8% and 24.3% of the patients, respectively, with no difference between the two groups. Table 2 reports the main results, and Figure 3 shows the trend of PED access and its incidence during the years of the study period.

### 3.3. Psychomotor Agitation

**Figure 4:**
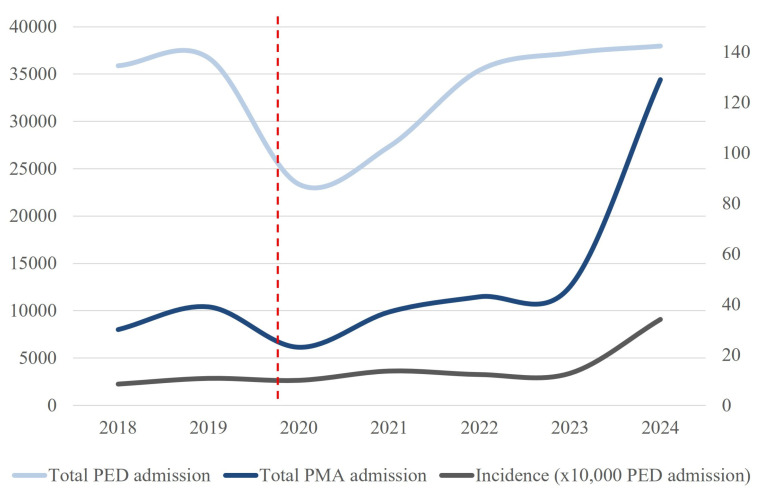
Trends in pediatric emergency department (PED) admissions for psychomotor agitation (PMA), total PED visits, and incidence rates over time. The light blue line represents the total number of PED admissions from 2018 to 2024. The dark blue line indicates the total number of psychomotor agitation. The gray line illustrates the incidence of PMA, calculated as the number of cases per 10,000 PED admissions. The vertical red dashed line indicates the onset of the COVID-19 pandemic (2020), followed by a sharp rise in both PMA-related visits and incidence, particularly in recent years.

A total of 348 PMA accesses were counted. PMA cases increased from 77 pre-COVIDcases to 271 post-COVID cases, with the incidence increasing from 9.4 to 17.8 per 10,000 visits (*p* < 0.0001). Male representation decreased (62.3% to 23.2%; *p* < 0.0001), whereas median age showed a non-significant increase (11.8 to 12.8 years; *p* = 0.06). Hospitalization and pharmacological sedation rates did not differ significantly between the periods. However, the concomitant heteroaggressive behavior increased significantly from 14.3% to 39.8% (*p* < 0.0001). Table 3 reports the main results, and Figure 4 shows the trend of PED accesses and its incidence during the years of the study period.

## 4. Discussion

Although the COVID-19 pandemic has markedly exacerbated the pediatric mental health crisis, several studies have shown that PED visits for psychiatric conditions in children and adolescents were already on the rise in the decade preceding the pandemic. In this context, COVID-19 acted as an amplifying factor, accelerating a pre-existing upward trend and exposing the structural vulnerabilities of pediatric mental health systems [1,6,7,16,17]. Our findings highlight this multifaceted impact of the COVID-19 pandemic on pediatric psychiatric emergency care in Italy, consistent with previous findings from literature worldwide [1,2,11,12]. PED utilization has increased disproportionately for psychiatric issues, and the dramatic rise in SIB and PMA observed in our study mirrors data from multiple centers reporting surges in these diagnoses following the onset of the pandemic [1,5,10]. The dramatic rise in SIB is consistent with the results of a meta-analysis that revealed increased suicide-related PED visits, particularly among adolescent females [1,6]. Our data also indicate an increase in female presentations and repeated PED use, suggesting systemic challenges in post-crisis outpatient care [1,6]. Given that suicide is the second leading cause of death in people aged 10–24 years, the need for proactive suicide screening in primary care settings should be emphasized, even in low-risk presentations, supporting the broader implementation of universal screening protocols [2,9,13,18]. The reduction in male patient representation across the AD and PMA categories may reflect shifting diagnostic patterns or gender-related stress responses, warranting further investigation [2,3,4,7]. Moreover, the stability of hospital admissions and pharmacologic intervention rates despite increasing patient volumes may indicate effective triage systems and PED management, although it raises concerns about staff burden and resource allocation. Furthermore, it may be possible that the stable admission rate is derived from a lack of availability of beds in a dedicated psychiatric ward, with a consequent increase in the length of stay in the PED.

AD visits also increased significantly, consistent with trends observed in Asian and Middle Eastern populations, where anxiety- and stress-related PED presentations increased during the pandemic [3,4,5,6,7]. These findings underscore the wide spectrum of pediatric psychiatric presentations and the need for emergency physicians to recognize early signs of escalating distress [2,8].

The observed age distribution and symptom severity did not align with previous reports that identified increasing presentations among preteens, particularly females with SIB [1,3,4,6]. In the SIB and PMA groups, we noticed a trend towards a higher age of presentation in the post-COVID period; however, this may be explained by the fact that we have increased the maximum age of acceptance in the PED from 14 to 15 years to improve patient management and to lighten the patient load in adult psychiatry units.

The extended length of stay (LOS) for children undergoing pharmacological or physical restraint is consistent with multiple studies citing psychiatric boarding as a systemic issue [12,13,14]. These prolonged stays are clinically and ethically problematic given their association with delayed treatment, staff strain, and increased resource consumption [15].

Unfortunately, we had incomplete data on the PED LOS. Thus, we cannot provide a conclusion on this matter, and we limit ourselves to supporting what has been suggested by other authors, who have emphasized the ethical and clinical challenges posed by prolonged boarding and delayed psychiatric placement [12,13,15]. This is corroborated by earlier findings reporting suboptimal care pathways in emergency psychiatric settings where new strategies such as teleconsults are warranted [5,6].

The notable increase in PMA cases and heteroaggressive behaviors postpandemic aligns with previous studies reporting a lack of consensus in PED management strategies for pediatric agitation [8,14,15]. Structured protocols and interdisciplinary team training have been advocated to manage acute behavioral crises in children [8,14]. Likewise, pharmacologic interventions must balance safety and efficacy and should follow a multimodal approach, including non-pharmacological and de-escalation methods [8,14,15,19,20]. Recent reviews have recommended timely, effective, and tailored sedation strategies based on age, severity, and risk factors [15,19,20].

Thus, from a treatment perspective, our pharmacological patterns align with the current consensus and emerging strategies [14,15,19,20]. Benzodiazepines and second-generation neuroleptics remain the most commonly used agents for acute PMA and appear to be safe and welltolerated [14,15,16,19,20,21,22]. Emerging pharmacological strategies, including the use of intranasal sedatives such as dexmedetomidine and ketamine, have gained attention as potential tools to manage acute agitation in PEDs with fewer adverse effects [14,23,24]. Our experience, although limited to only fivecases, supports these indications. Other recent reports have shown promising outcomes for intranasal dexmedetomidine in pediatric PMA [24]. Both agents are increasingly favored for their ease of administration and safety profile, although pediatric-specific protocols for psychiatric emergencies remain inconsistent across institutions [18,24]. We did not identify a greater use of pharmacological sedation in our patients, except for a slightly increased use of drugs in SIB admissions in the post-COVID period, probably due to concomitant ADs. These data suggest that there was an increase in PED accesses but without an increase in severity. Notably, we did not notice any adverse events. In addition, we did not observe an increase in the use of polytherapy, which further corroborates the non-increased severity of AD or PMA episodes. The main drug used in our PED wasdelorazepam, intranasal, oral, or intramuscular, depending on the patient’s cooperation and containment [16,22]. As a second-line treatment, a neuroleptic was mainly used, specifically chlorpromazine and less frequently clotiapine, both orally and intramuscularly [22]. As stated before, in five cases, all in the post-COVID period, intramuscular or endonasal ketamine was used as a third line of treatment due to failure of benzodiazepines and neuroleptics, with no adverse effects recorded. Emerging evidence for the use of the endonasal route for the administration of sedative therapies for severe agitation is promising, both in terms of efficacy, which is faster than intramuscular administration, and in terms of safety, with a lower risk of needlestick injury to the patient [22,23,24].

However, evidence remains heterogeneous and standardized treatment protocols are lacking in pediatric emergency settings [15,18,19]. There is still significant variability in medication protocols, particularly for intranasal formulations [18,24]. Our results support the need for standardized evidence-based guidelines tailored to the pediatric population [15,19,23].

Beyond these findings, it should be highlighted that there has been a steady rise in PED psychiatric visits over the last decade and a call for improved psychiatric training in PEDs [6,11,12]. Our data also suggest that there is an increase in recurrent PED users with 2+ accesses per patient, in agreement with previous analyses that revealed that more than one-quarter of children returned to the ED within 30 days after psychiatric hospitalization, often because of inadequate outpatient continuity [9,10]. This reinforces the call for improved transitional care and follow-up strategies, as well as improving tele-psychiatry consultations and psychiatric primary care services to prevent unplanned PED utilization for psychiatric re-exacerbation [5,6,8,10].

There is an urgent need for solutions to improve the care of youth mental health emergencies, and these should include better coordination between mental health primary care, PEDs, and telepsychiatry services and improvements inboth the professional capacities of PED physicians and structures [6,8,9,13].

Specific strategies to improve transitional care and reduce unplanned PED revisits may include structured follow-up planning at discharge, family-based psychoeducation, and active case management. Telepsychiatry networks, particularly school-linked and community-based services, can bridge gaps in mental health access. Standardized post-discharge protocols, early referral to child and adolescent mental health services, and collaborative care models with primary care pediatricians are also recommended [5,6,8,9,10].

### Limitations

This study had several limitations. First, it was based on retrospective data from a single tertiary care center, which may limit the generalizability of the findings to other settings. Moreover, the manual reviews of patients’ charts, even if well conducted, can determine biases relating to subjectivity and data reliability. Second, clinical documentation variability and potential underreporting in electronic medical records could have influenced the data accuracy. Third, the study did not evaluate the long-term outcomes postdischarge. Lastly, although we identified pharmacological trends, the study did not explore the detailed long-term safety or efficacy outcomes of each agent.

Future studies should aim to validate our findings through multicenter designs and longer-term follow-up. The implementation of standardized severity scores and prospective documentation of pharmacological responses and adverse events would strengthen future analyses.

## 5. Conclusions

To our knowledge, this is the first study in Italy to provide a detailed condition-specific analysis of pediatric psychiatric emergency visits. Our study highlights and confirms the increasing burden of pediatric psychiatric emergencies in the post-pandemic era, especially AD, SIB, and PMA. The current findings contribute to a growing body of evidence supporting the urgency of systemic reforms to safeguard child and adolescent mental health in the wake of global crises. Moreover, these findings align with the current epidemiological trends and support prior calls for expanded pediatric mental health services and emergency protocols tailored to complex psychiatric presentations.

The variability in medication practices and absence of pediatric-specific guidelines underscore the urgent need for consensus-driven protocols, and a multidisciplinary approach, including early intervention, standardized sedation protocols, and improved outpatient continuity, may alleviate pressure on PED services.

Future directions should include the development of multicenter pediatric sedation guidelines, expanded integration of psychiatric services and dedicated de-escalation areas into the PEDs, greater integration of digital mental health screening tools in primary care settings such as schools, referral centers, and general practice offices, and structured discharge planning and linkage to outpatient services to reduce return visits.

Ultimately, a coordinated and multidisciplinary approach is essential to address the evolving landscape of pediatric psychiatric emergencies and optimize care outcomes across the continuum.

## Figures and Tables

**Table 1:** Clinical and demographic characteristics of pediatric emergency department (PED) visits for anxiety disorders, comparing pre-COVID and post-COVID periods. COVID: CoronaVirus Disease 2019; PED: Pediatric Emergency Department.

	Total Accesses *n* = 474	Pre-COVID Accesses *n* = 145	Post-COVID Accesses *n* = 329	*p*
Gender, male (%)	163 (34.4)	50 (34.5)	113 (34.3)	0.97
Median age in y (IQR)	12.5 (11.0–13.8)	12.3 (10.6–13.8)	12.6 (11.2–13.8)	0.38
Incidence ×10,000 PED accesses	20.2	17.7	21.6	0.018
Admission, yes (%)	74 (15.6)	21 (14.5)	53 (16.1)	0.65
Drugs, yes (%)	68 (14.3)	21 (14.5)	47 (14.3)	0.95
Recurrent PED users, yes (%)	49 (10.3)	17 (11.8)	32 (9.8)	0.51

**Table 2:** Clinical and demographic characteristics of pediatric emergency department (PED) visits for self-injury behavior, comparing pre-COVID and post-COVID periods. COVID: CoronaVirus Disease 2019; PED: Pediatric Emergency Department.

	Total Accesses *n*= 260	Pre-COVID Accesses *n*= 30	Post-COVID Accesses *n* = 230	*p*
Gender, male (%)	32 (12.3)	7 (23.3)	25 (10.8)	0.05
Median age in y (IQR)	13.6 (12.8–14.4)	13.2 (11.1–13.9)	13.6 (13.0–14.4)	0.01
Incidence ×10,000 PED accesses	11.1	3.6	15.1	<0.0001
Admission, yes (%)	215 (82.6)	25 (83.3)	190 (82.6)	0.92
Drugs, yes (%)	32 (12.3)	0 (0)	32 (13.9)	0.03
Concomitant suicide attempt, yes (%)	63 (24.3)	10 (33.3)	53 (23.0)	0.21
Concomitant cutting, yes (%)	88 (33.8)	10 (33.3)	78 (33.9)	0.94
Concomitant mood swing, yes (%)	41 (15.7)	3 (10)	38 (16.5)	0.35
Recurrent PED users, yes (%)	64 (24.6)	1 (3.4)	63 (27.4)	0.004

**Table 3:** Clinical and demographic characteristics of pediatric emergency department (PED) visits for psychomotor agitation, comparing pre-COVID and post-COVID periods. COVID: CoronaVirus Disease 2019; PED: Pediatric Emergency Department.

	Total Accesses *n* = 348	Pre-COVID Accesses *n* = 77	Post-COVID Accesses *n* = 271	*p*
Gender, male (%)	111 (31.9)	48 (62.3)	63 (23.2)	<0.0001
Median age in y (IQR)	12.6 (10.9–13.8)	11.8 (10.2–13.5)	12.8 (11.0–13.9)	0.06
Incidence ×10,000 PED accesses	14.8	9.4	17.8	<0.0001
Admission, yes (%)	176 (50.5)	37 (48.0)	139 (51.3)	0.61
Drugs, yes (%)	131 (37.6)	27 (35.0)	104 (38.4)	0.59
2+ Drugs, yes (%)	31 (8.9)	6 (7.8)	20 (7.4)	0.72
Concomitant heteroaggression, yes (%)	119 (34.2)	11 (14.3)	108 (39.8)	<0.0001
Recurrent PED users, yes (%)	96 (27.5)	19 (24.7)	77 (35.5)	0.20

## Data Availability

The datasets used and analyzed in this paper are available from the corresponding author on reasonable request.

## Abbreviations

The following abbreviations are used in this manuscript:

COVID-19	CoronaVirus Disease 2019
PED	Pediatrics Emergency Department
AD	Anxiety Disorders
SIB	Self-Injury Behaviors
PMA	Psychomotor Agitation
LOS	Length of Stay
